# Regulators, Pivotal Clinical Trials, and Drug Regulation in the Age of COVID-19

**DOI:** 10.1177/0020731420979824

**Published:** 2020-12-21

**Authors:** Joel Lexchin, Janice Graham, Matthew Herder, Tom Jefferson, Trudo Lemmens

**Affiliations:** 1 Faculty of Health, York University, Toronto, Ontario, Canada; 2 Faculty of Medicine, Dalhousie University, Halifax, Nova Scotia, Canada; 3 Shulich School of Law, Health Law Institute, Halifax, Nova Scotia, Canada; 4 Department of Continuing Education, University of Oxford, Oxford, UK; 5 Faculty of Law Toronto, University of Toronto, Toronto, Ontario, Canada

**Keywords:** COVID-19, drug regulators, European Medicines Agency, Food and Drug Administration, Health Canada, pivotal trials

## Abstract

Medicine regulators rely on pivotal clinical trials to make decisions about approving a new drug, but little is known about how they judge whether pivotal trials justify the approval of new drugs. We explore this issue by looking at the positions of 3 major regulators: the European Medicines Agency, Food and Drug Administration, and Health Canada. Here we report their views and the implications of those views for the approval process. On various points, the 3 regulators are ambiguous, consistent, and demonstrate flexibility. The range of views may well reflect different regulatory cultures. Although clinical trial information from pivotal trials is becoming more available, regulators are still reluctant to provide detailed information about how that information is interpreted. As medicines and vaccines come up for approval for treatment of COVID-19, transparency in how pivotal trials are interpreted will be critical in determining how these treatments should be used.

Pivotal clinical trials are key in the regulatory approval of medicines and vaccines, and how regulatory agencies interpret these trials can ultimately influence whether their decisions are accepted by the general public. According to Health Canada, pivotal trials have “high scientific quality, which provide the basic evidence to determine the efficacy, properties, and conditions of use of the drug.”^1^ The U.S. Food and Drug Administration (FDA) definition is similar.^2^

Regulatory agencies rely on “pivotal trials” to provide evidence of a product’s effectiveness and harm/benefit, but scant research exists on how regulators interpret and use such trials in their final decisions and how their decisions reflect regulatory culture. For example, regulation from the European Parliament outlining procedures for the authorization of medicinal products and establishing the European Medicines Agency (EMA) makes no mention of the term pivotal trials.^3^ Yet, variable interpretation by regulatory agencies of the evidence in pivotal trials is evident. For example, 12 of 37 medicines with novel mechanisms of action approved first in Europe and/or Canada had their initial FDA submissions rejected for safety reasons.^4^

As a group concerned about regulatory policy—how it is developed and applied—we were concerned by our inability to understand the relationship between regulatory agencies and the pharmaceutical industry when it came to pivotal trials and how the agencies negotiated the use of these trials. We were particularly interested in questions of how decisions were made about the designation of pivotal trials and how evidence from them was used in the regulatory process. Despite an extensive search of the websites of 3 major regulators—the EMA, the FDA, and Health Canada—we were unable to find any policy documents that dealt with these issues.

In the absence of any policy literature about pivotal trials, we contacted the EMA, FDA, and Health Canada by email to obtain their points of view on 4 specific questions: Who designates a clinical trial as pivotal? When is the designation made? How do they use the term “failed trial”? and How are decisions made when trials are positive and negative? The regulatory practices of these 3 agencies carry global implications; their decisions about whether pivotal trial data justify approving a new drug are often relied upon by the three-quarters of the World Health Organization membership with resource-challenged regulatory systems.^5^ Health Canada was contacted through its Therapeutic Products Directorate, the branch that approves traditional small molecules. The FDA was contacted through its Office of Media Affairs and the EMA through its Media and Public Relations Service. We did not seek ethical approval because we were contacting the agencies themselves and not specific individuals within the agencies.

Here we discuss the positions they take and what those positions mean for drug regulation. Table 1 provides the complete verbatim replies from each agency.

**Table 1.** Regulators’ Answers to Questions About Pivotal Clinical Trials.

Question	Health Canada	Food and Drug Administration	European Medicines Agency
Who designates a clinical trial as “pivotal”? Is it [name of the agency] or the company filing the New Drug Submission or is it both in consultation?	The sponsor usually identifies which of their studies are pivotal in their submission. During submission review, Health Canada might also look to other studies in the submission as being pivotal. For example, a sponsor might not consider a food effect study to be pivotal. However, Health Canada considers a food effect study to be pivotal when the study results in specific numbers or wording describing a food effect anywhere in the Product Monograph, such as an impact on PK parameters (AUC, Cmax).	The term “pivotal” does not show up in regulations, but its general meaning to people is clear enough: It is the trial or trials that will be, or could be, the basis for our reaching a conclusion that there is “substantial evidence of effectiveness,” the statutory and regulatory standard for approving a drug. That would also mean that the FDA considers it (or them) an “adequate and well-controlled investigations,” which is the only basis for accepting a study as supporting effectiveness. A pivotal trial presents the most important data used by FDA to decide whether to approve a drug.	From a regulatory perspective, we would consider a clinical trial as pivotal if it provides robust confirmatory evidence of a clinically relevant effect in the target population with an acceptable safety profile, taking available therapies into account. A pivotal study forms the basis for the regulatory benefit/risk assessment. In a marketing authorization application (MAA), the sponsor may define which studies they consider pivotal, but the Agency makes its own assessment and decision on which studies it considers pivotal for the benefit/risk assessment. The sponsor may seek scientific advice from the Agency (see below) on how a study should be designed to most likely generate pivotal data for the regulatory assessment and thus be considered pivotal.
Does the designation of a pivotal clinical trial happen before the trial begins or after the trial is completed?	“Pivotal” is a designation for health product submissions; however, the level of evidence (either confirmatory or exploratory) should be outlined in the study protocol prior to its commencement. Confirmatory trials should have pre-specified primary endpoint(s) and have methods in place to adequately control for type I error (i.e., ensure a sufficiently low false-positive rate). Confirmatory evidence from clinical trials is generally required to support the proposed indications. However there may be other factors to consider, such as severity of disease, the size of the patient population, lack of other treatment options, safety concerns compared to other treatments, etc., when assessing the entirety of the evidence of a submission that may lead to more reliance on the results of exploratory trials. The designation of a pivotal trial is generally given to confirmatory trials.	A sponsor usually identifies the trials intended to provide evidence of effectiveness before the trial commences, and often discusses this with the FDA to see that we agree on the design, endpoints, analyses, etc., but sometimes, earlier trials not intended to be definitive provide unexpectedly strong results, or show effects on a potential surrogate endpoint, such that the sponsor comes to believe that the trial or an effect on a surrogate endpoint can be relied on as a basis for approval.	Preferably, a study should be prospectively designed to be pivotal (provide pivotal evidence for the regulatory benefit/risk assessment), and most often, this is the case. During the regulatory assessment of the results, the Agency may conclude that the trial does not provide pivotal evidence. Still, there may be exceptional instances where a study is not originally designated as pivotal, but the results provide pivotal evidence because they are compelling. In this situation, the trial may thus be considered pivotal by the sponsor and the Agency.
Does [name of the agency] use the term “failed trial”? If so, how is that term defined?	Yes, we do. A failed study is a study that failed to meet statistical significance in its primary endpoint(s). Other endpoints in the study may have shown statistical significance; however, these results would be considered exploratory or hypothesis-generating (i.e., requiring another study to confirm the results). It is possible for a study to still be a successful study, even if one or more primary endpoints failed, provided one or more still passed and there was adequate control of type I error.	The term “failed trial” is not defined in FDA’s regulations, but is widely recognized to mean a trial that did not succeed in showing the intended effect (generally, a statistically significant effect on the intended primary endpoint). There are degrees of “failure.” A trial that leans in a favorable direction but does not quite meet the intended level of statistical significance may not support approval, but may encourage further study. Trials that do not show an overall effect may have subgroups or other endpoints that suggest potential favorable results and may encourage further study with a somewhat different design. These “failed” studies are not necessarily complete failures. The term is also ambiguous as to whether it means that the study failed to show an effect that was probably there or showed that the anticipated effect was just absent.	Officially, no, as the term is ambiguous. For example, an adequately designed trial may not succeed to show an effect on the primary endpoint because the drug is not effective or shows unacceptable adverse effects. From a regulatory perspective, this is not a “failed trial” as it generates important information for the benefit/risk assessment. On the other hand, a trial may succeed on the endpoints and not identify safety issues, but may not allow a benefit/risk assessment due to design flaws. Thus from a regulatory perspective, trials should be judged based on how they generate adequate data for the benefit/risk assessment.
If the pivotal trials are positive but other trials are either negative or failed trials (assuming that the latter term is used), how does [name of the agency] take the negative and failed trials into account in deciding whether to grant marketing authorization?	Both positive and negative (failed) trials can be part of a submission when assessing the entirety of the evidence of a submission. For example, a failed study could still provide useful safety information, though any discordance between studies regarding efficacy results would have to be closely examined to determine what the potential causes were. The acceptability of exploratory results for supporting the efficacy of an indication depends on several factors, some of which are listed above in the response to question 2. In general, if the pivotal trials are positive, but the supportive trials are negative (i.e., failed to meet statistical significance in the primary endpoint), Health Canada will expect the sponsor to provide a rationale to explain this discordance. Following thorough submission review, a Notice of Compliance may or may not be granted (i.e., an interim regulatory decision, such as a Notice of Deficiency or Notice of Noncompliance, may be issued to allow the sponsor to provide further evidence in support of the proposed indication).	The FDA does not expect that all trials, even of effective drugs, will succeed. In some therapeutic areas, it is common for well-conducted trials of effective drugs to not show an effect, perhaps because of patient variability or other unrecognized reasons. This is very common in depression trials, where about half of trials of effective agents do not show the effectiveness. A negative trial does not undermine the positive trials as a general rule, although a large number of them could do so, raising the possibility that the positive trial was a chance effect. This would be a greater concern where there was only one positive study.	This is a complex issue and very much an assessment issue. Other “negative or failed” trials may be with the same medicinal product, the same class of products, or with a different class of products in the same therapeutic area. There are indeed therapeutic areas with a history of failed studies. If there are other “negative or failed” trials, this is taken into account for the benefit/risk assessment. The sponsor would need to explain/justify why their medicinal product with its trial designs, target population, etc., is “successful” where others have failed. Special attention will be paid to the preclinical studies, tolerance studies, dose-finding, and other phase II studies. Results have to be particularly compelling with respect to internal and external validity, clinical relevance, statistical significance, data quality, and internal consistency. Strong confirmatory evidence would be required, such as 2 pivotal trials or one pivotal trial with a high degree of statistical evidence.

## Regulators’ Positions on Pivotal Trials

### The Designation of a Clinical Trial as Pivotal

In theory, regulators should have no predetermined economic interest as to which trial should be considered pivotal, whereas the company sponsoring the trial does. If companies make the designation, they are incentivized to maximize the chance of positive results through trial design, collection of data, and its final analysis, leading to results and conclusions that more likely favor the sponsor.^6^

Health Canada’s position is that the company usually designates a trial as pivotal in its regulatory submission. Under certain circumstances, the agency can determine that a trial is pivotal without a designation from the company. The EMA takes essentially the same position as Health Canada. The FDA emphasized that the term “pivotal trial” is not present in its regulations, but that it can be understood to refer to one or more trials that form the basis of its conclusion of substantial evidence of effectiveness. However, it is not clear if the FDA or the company makes the decision about which trials are pivotal.

### The Timing of Making the Decision About Whether a Trial Is Pivotal

The designation of a trial as pivotal should be made in the protocol *before* the trial begins in order to remove any regulatory ambiguity about the timing of results that are generated. This a priori decision may be particularly relevant in the case of urgent research during emergencies such as the COVID-19 pandemic. For example, it is unclear how the early termination of the National Institutes of Health-sponsored study on remdesivir before there was definitive data on its effect on mortality^7^ affected its presumptive designation as a pivotal trial.^8^

Health Canada differentiates between exploratory and confirmatory trials and believes that the level of evidence should be outlined in the protocol prior to commencement of the study—that is, that the decision should be made a priori. It equates pivotal studies with confirmatory ones, but allows that other factors may lead to more reliance on the results of exploratory trials.

The EMA’s position is also that a trial should prospectively be designated as pivotal and that this designation is typically made by the company sponsoring the trial, although the company’s decision may be made after meetings between the agency and the company. Like Health Canada, its position is that in exceptional instances, trials not originally identified as pivotal may provide compelling evidence or alternatively that trials that were initially considered pivotal could be downgraded in status during the evaluation process.

The FDA’s position is also that the sponsor usually identifies the trials intended to provide evidence of effectiveness before the trial commences, and the trial is often designed based on discussions between the company and the FDA. According to the FDA, effects on surrogate endpoints in earlier trials can sometimes provide strong enough evidence for approval—that is, these trials can achieve pivotal status.

### Failed Trials

A “failed trial” is usually interpreted as one whose results fail to show that the drug is efficacious due to some failure of assay sensitivity—for example, a flaw in sample selection, outcome assessment, or clinician adherence.^9^ It is important to understand how regulators distinguish between failed and negative trials. If regulators decide that a trial failed, the results can be discounted, whereas a decision that a trial was negative may mean that the drug will not be approved.

According to Health Canada, a “failed study” is one where the primary endpoint(s) does not achieve statistical significance. Although other endpoints might show statistical significance, results are considered exploratory or hypothesis-generating and need to be confirmed in a subsequent study. The EMA does not use the term and considers it “ambiguous.” In its view, even an adequately designed trial may not show efficacy if there are unacceptable side effects or no clinical benefits. In either case, the EMA does not necessarily consider this a failed trial if it has generated useful information.

The FDA’s position is that the term “failed trial” is not defined in its regulations, but that it is commonly understood to mean a trial that did not succeed in showing the intended effect. It also considers the term ambiguous because it could mean that the study failed to show an effect that was probably there or it showed that the anticipated effect was just absent. Like the other 2 regulators, the FDA felt that so-called failed trials could generate useful information.

### Decision-Making When Some Pivotal Trials Are Positive and Others Are Negative

Typically, regulators require 2 positive pivotal trials to approve a new drug, although that requirement has been somewhat eroded. Between 1995 to 2017, the proportion of new drugs and biologics approved by the FDA using 2 pivotal trials declined from 81% to 53%.^10^ This trend in the decline of using 2 pivotal trials as the basis for approval is not unique to the FDA. A 2019 study documented the approval of 23 novel therapeutic drugs between 2012 and 2016 by both the FDA and the EMA based on a single pivotal trial.^11^

There is no legislative requirement in the United States regarding what percentage of trials need to be positive. According to a legal analysis, “in theory, a drug sponsor could simply run clinical trials in sequence, stopping only after 2 positive trials have been accumulated.”^12^ Using those 2 trials, the FDA could satisfy the criterion of substantial evidence of efficacy, notwithstanding the other negative trials. Negative trials appear not to hinder approval; about half of the antidepressant trials submitted to the FDA between 1987 and 2004 for regulatory approval of 12 drugs were negative.^13^ Similarly, in Canada, companies only have to submit “substantial evidence of the clinical effectiveness of the new drug,” with no minimum number of trials specified and no mention of negative trials.^14^

The position of Health Canada is that it would expect the sponsor to provide an explanation if the pivotal trials were positive but the supportive (exploratory) trials were negative, and it may require further supportive information. The EMA calls this situation a complex assessment issue; it requires the sponsor to explain the discrepancy in the results and strong confirmatory evidence would be necessary for approval. According to the FDA, not all trials are expected to be positive. The FDA takes the position that, as a general rule, a negative trial does not undermine the positive trials, although a large number of them could do so, raising the possibility that the positive trial was a chance effect.

## Regulators, Pivotal Trials, and Drug Regulation

The positions of the regulators about pivotal trials provide valuable insights into their views on drug regulation in general.

### Ambiguity

The 3 regulators often take ambiguous positions, creating a discretionary space. This ambiguity may be an artifact of regulators’ historical aversion to disclose information used in decision-making. Until relatively recently, both the EMA and Health Canada were unwilling to make public clinical trial reports filed by drug companies, and the FDA still does not do so.^15^

### Similarities

The positions taken by all 3 regulators were similar in many respects. Again, this should not be surprising. All 3 are members of the International Council for Harmonisation of Technical Requirements for Pharmaceuticals for Human Use, an alliance of regulatory authorities and pharmaceutical industry associations that together participate in the development and implementation of the council’s standards. There are also substantial interconnections among the 3 regulators based on Mutual Recognition Agreements.^16^

### Distinct Therapeutic Cultures

The positions may well reflect distinct therapeutic cultures in the different regions. These “therapeutic cultures arise from networks of actors that produce regulatory policy, determine testing standards, and ultimately decide on market access for new drugs.”^17^ Variations in these networks can be seen in the different ways that agencies compose their advisory committees, how they structure their interactions with industry, and the extent to which they integrate patients into their processes.^18–20^ Two comparisons of how the FDA and the EMA make decisions on oncology drugs found that the 2 manage uncertainty differently. The conclusion from one study was that the FDA is “more open to take risks and base approval on less robust data in order to guarantee quicker access to anticancer medications.”^21^ The second study did not find any data showing that the FDA took more risks, but did conclude that the 2 agencies approached risk differently.^22^ Both studies illustrate that informal factors, while secondary to the data in driving decisions, play an important role in the drug-regulation process.

### Flexibility and Transparency

Finally, drug regulators are flexible in how they interpret clinical evidence—that is, in their use of discretionary power. Flexibility is desirable and necessary because the data require interpretation and the balance between benefit and harm can be hard to determine.^23^ However, how this flexibility is expressed should be transparent and justified so that both health care providers and the public understand the rationale for the decisions regulators make. Yet, recent changes to the FDA’s review process—replacing individual scientific reviews that often contain a wealth of information that is not available elsewhere, as well as conflicting interpretations of the evidence,^24^ with one “integrated review”—stand to further obscure regulatory decision-making.^25^

The onset of the COVID-19 crisis has seen conflicting advice from the FDA. On March 28, 2020, it issued an emergency use authorization, based on case series data, giving doctors permission to prescribe hydroxychloroquine off-label for patients with COVID-19.^26^ Less than a month later, the FDA qualified that authorization and said the drug should only be prescribed to patients who are in the hospital or enrolled in a clinical trial.^27^

Differences between regulatory agencies are also apparent during the COVID pandemic, despite mutual agreements. For example, the EMA did not authorize the use of hydroxychloroquine for unapproved indications.^28^ Different regulatory instruments within the 3 agencies result in different mechanisms to approve remdesivir “conditionally” (Health Canada) or pre-approval as an emergency use authorization (FDA).^29^ Most recently, the FDA took extra due diligence in examining safety data, restarting the Phase III Oxford-Astra Zeneca COVID vaccine clinical trial AZD1222 fully 6 weeks later in the United States (October 23) compared to the United Kingdom (September 14) after it was stopped on September 6 due to an adverse event following immunization.^30^

## Conclusion

The decision to approve a new drug by a regulatory agency is a hybrid of the scientific evidence in pivotal trials and cultural practices that reflect national values and structures. To be fully accountable to the public, regulators must help us understand how they integrate the two. Taking steps to disclose judgment calls about why a trial is considered pivotal and how the evidence from the trial is interpreted would markedly improve public understanding of regulatory decision-making and enhance transparency and accountability.
